# Supplementary material for: Closed-Loop Recycling of Poly(vinyl butyral) Interlayer Film via Restabilization Technology
Source: Polymers (Basel). 2025 Jan 24;17(3):317. doi: 10.3390/polym17030317 (PMC11820937; doi:10.3390/polym17030317)
Supplement: Supplementary file 1 [file polymers-17-00317-s001.zip › polymers-3411581-supplementary.pdf]

SUPPLEMENTARY INFORMATION

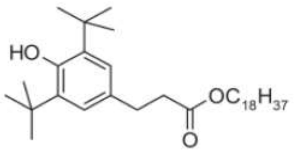

octadecyl 3-(3,5-di-tert-butyl-4-hydroxyphenyl)propionate

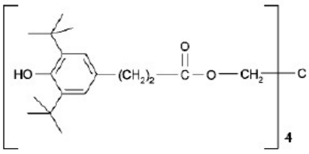

Pentaerythritol tetrakis(3-(3,5-di-tert-butyl-4-hydroxyphenyl)propionate)

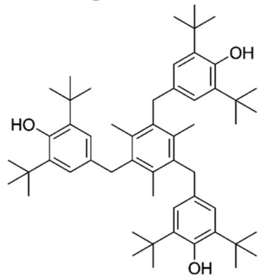

1,3,5-trimethyl-2,4,6-tris(3,5-di-tert-butyl-4-hydroxybenzyl)benzene

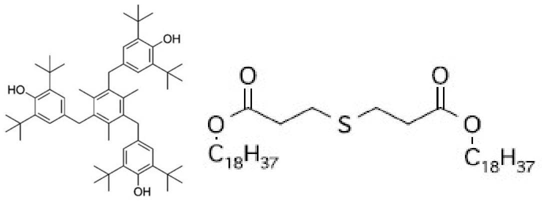

AO-3 + octadecyl 3-[[3-(dodecyloxy)-3-oxopropyl]thio]propionate (1:1)

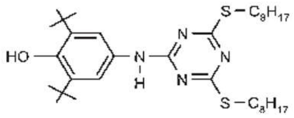

4-[[4,6-bis(octylsulfanyl)-1,3,5-triazin-2-yl]amino]-2,6-ditert-butylphenol

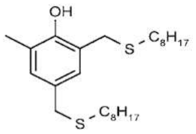

2-methyl-4,6-bis[(octylthio)methyl]phenol

Figure S1 : Chemical structures of the incorporated antioxidants (AO).

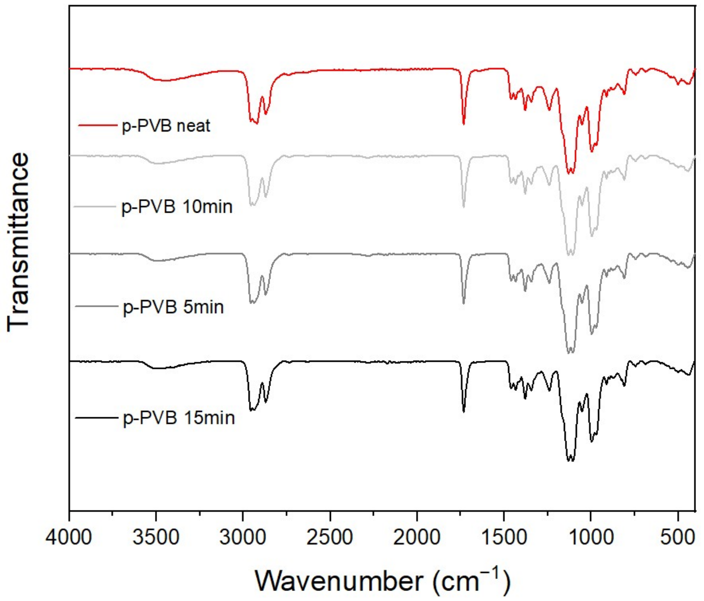

Figure S2: FT-IR of unstabilized p-PVB (neat, 5, 10, 15 min at 150°C, 40 rpm in internal mixer)

Table S1: MFR, OOT and YI for the neat, un-stabilized and stabilized with 0.3% of different AOs (AO 1-6) for u-PVB & p-PVB in the critical condition of 10 minutes in the internal mixer.

Samples	MFR* (g/10min)	OOT (°C)	YI (%)
**u-PVB samples**			
Neat u-PVB	0.40 ± 0.02	219 ± 1.5	-11.17 ± 0.13
Kneaded	1.49 ± 0.10	206 ± 1.7	-6.09 ± 0.09
AO-1	1.13 ± 0.01	235 ± 1.4	0.15 ± 0.48
AO-2	0.87 ± 0.03	239 ± 0.8	-4.35 ± 0.15
AO-3	1.06 ± 0.04	242 ± 1.1	-1.14 ± 0.24
AO-4	0.37 ± 0.05	259 ± 0.9	0.69 ± 0.58
AO-5	0.64 ± 0.01	248 ± 1.4	7.41 ± 0.33
AO-6	0	202 ± 1.1	20.96 ± 2.07
**p-PVB samples**			
Neat p-PVB	1.94 ± 0.05	212 ± 1.8	-5.75 ± 0.71
Kneaded	4.28 ± 0.30	208 ± 1.6	-2.96 ± 0.68
AO-1	2.49 ± 0.10	229 ± 1.1	-2.28 ± 0.83
AO-2	2.56 ± 0.25	236 ± 1.3	-3.51 ± 1.41
AO-3	2.24 ± 0.12	242 ± 1.4	-4.57 ± 0.51
AO-4	2.10 ± 0.09	237 ± 1.4	-1.74 ± 0.30
AO-5	2.22 ± 0.08	250 ± 1.5	1.11 ± 0.52
AO-6	2.00 ± 0.12	230 ± 1.7	-3.65 ± 0.72

**u-PVB samples measured with 10 kg load, p-PVB samples with 2.16 kg.*

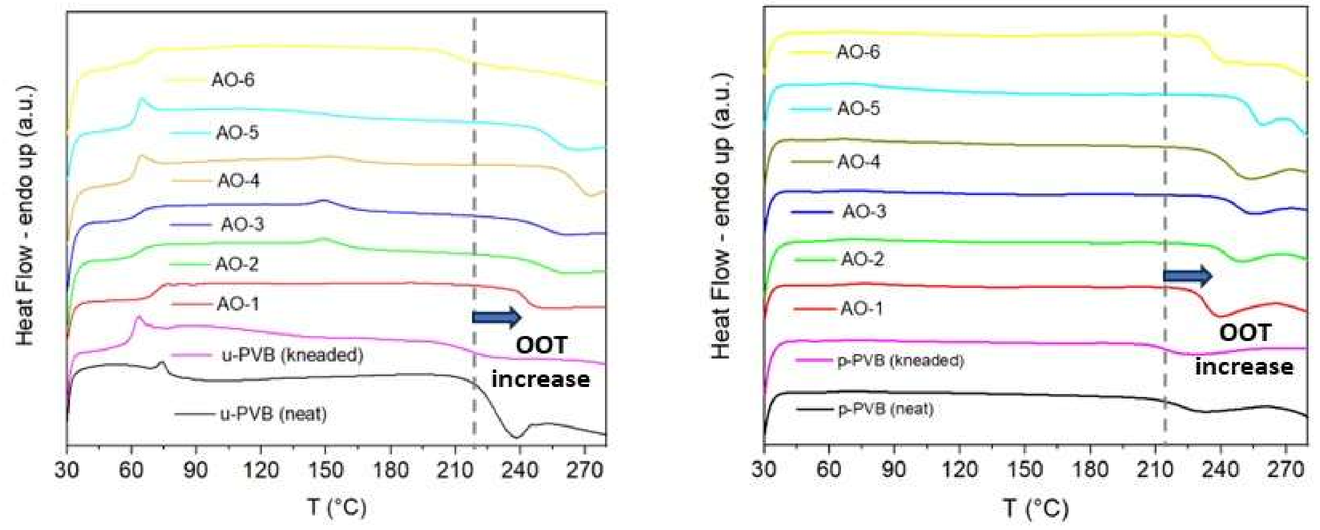

(**a**) (**b**)

Figure S3: Heating curves (DSC) indicating OOT for the six tested AO formulations in (**a**) u-PVB, (**b**) p-PVB.

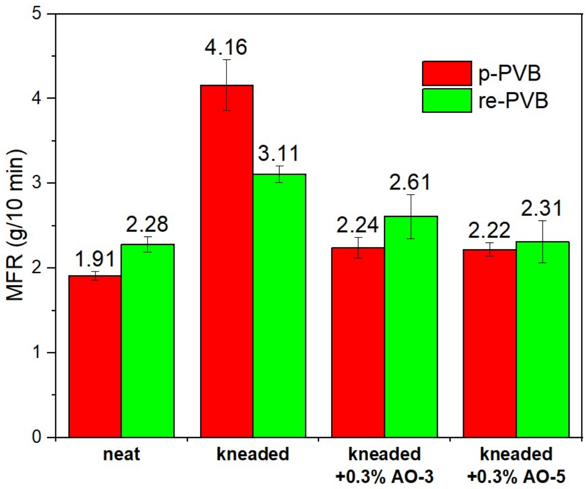

Figure S4: MFR for p-PVB & re-PVB unstabilized and stabilized with AO-3, AO-5 at critical condition

.
